# Equity of access to cardiac rehabilitation: the role of system factors

**DOI:** 10.1186/1475-9276-9-2

**Published:** 2010-01-21

**Authors:** Jennifer A Stewart Williams, Julie E Byles, Kerry J Inder

**Affiliations:** 1 Faculty of Health, University of Newcastle, Australia

## Abstract

**Background:**

When patient selection processes determine who can and cannot use healthcare there can be inequalities and inequities in individuals' opportunities to benefit. This paper evaluates the influence of a hospital selection process on opportunities to access outpatient cardiac rehabilitation (CR).

**Methods:**

A secondary data analysis was conducted on a cohort of inpatients (n = 2,375) who were all eligible for invitation to an Australian CR program. Eligibility was determined by hospital discharge diagnosis codes. Only invited patients could attend. Logistic regression analysis tested the extent to which individual patient characteristics were statistically significantly associated with the outcome 'invitation' after adjusting for cardiac disease and other factors.

**Results:**

Less than half of the eligible patients were invited to the CR program. After allowing for known factors that may have justified not being selected, there was bias towards inviting males, younger patients, married patients, and patients who nominated English as their preferred language.

**Conclusions:**

Health service managers typically monitor service utilisation patterns as indicators of access but often pay little attention to ways in which locally determined system factors influence access to care. The paper shows how a hospital selection process can unreasonably influence patients' opportunities to benefit from an evidence-based healthcare program.

## Background

Access to healthcare is a multifaceted notion encapsulating dimensions such as availability, affordability and accessibility. Access is characterised by a process of gaining entry into a system of care as well as the timely use of a service that delivers an appropriate level of care [1,2]. Healthcare services are typically evaluated by measuring utilisation or outcomes, but this approach does not necessarily identify all inequalities in access to care. Efficacious services that deliver health gains to many can increase health inequalities and inequities by restricting access for population sub-groups at the margins.

The concept of access to healthcare extends beyond service availability and includes various impediments that can prevent or limit service use. Barriers to access exist in many forms and can become embedded within healthcare systems. In addition to personal barriers, associated with individuals' needs, attitudes, beliefs and experiences, access impediments are associated with financial, geographic or organisational factors, such as levels of insurance, out of pocket costs, location and waiting times [3,4]. This paper shows how barriers to access can be inadvertently perpetuated by systems operating within local facilities.

While monitoring utilisation amongst those individuals who have already gained access to a service is important, it is also necessary to identify the extent to which individuals are excluded from opportunities to gain access. In their adaptation of the widely cited Behavioral Model of Health Service Utilisation [5,6] Aday and Andersen distinguished between the 'actual' use of services through which access is realised, and opportunities to gain entry to or use a service [6-8]. It is this second conceptualisation that is the focus of this paper. The aims are to show how a hospital selection process can unreasonably influence patients' opportunities to benefit from an evidence-based healthcare program.

### Setting

The evidence-based outpatient CR program at John Hunter Hospital (JHH) Newcastle, Australia, is used here to show the influence of a hospital selection process on opportunities to access outpatient CR. John Hunter Hospital is the principal tertiary referral hospital for the Hunter New England Region of New South Wales (NSW) of which there are over 800,000 residents. The Hospital adopted evidence-based clinical practice guidelines which endorsed the effectiveness of CR, recommending patients who received a diagnosis of acute myocardial infarction (AMI), unstable angina pectoris (UAP) or heart failure (HF) or had undergone coronary revascularisation, as being suitable candidates for the program [9,10].

Cardiac rehabilitation is a comprehensive tertiary prevention program of exercise, lifestyle education and counselling which is intended to minimise disability resulting from coronary heart disease (CHD) and prevent subsequent hospitalisation and death from cardiac related causes. The JHH program comprised a series of two-hour classes run by nurses and allied health professionals. The typical duration involved two or three weekly sessions conducted over a six to eight week period.

The CR selection process for participation in the JHH program comprised identification, assessment and invitation. Identification occurred when a patient was recognised by CR staff as part of a routine record alert system, or was actively referred to the CR team by a medical officer and documented on the CR database. Assessment occurred when a patient was reviewed by a CR nurse for suitability to attend outpatient CR and was recorded as being either invited or not invited, and invitation resulted when a patient was offered a CR program, and enrolled with a booked commencement date. Only invited patients could attend the program.

## Methods

A secondary data analysis was conducted on a cohort of 2,375 patients who were all eligible for invitation to the JHH CR program. The outcome 'invitation' was a dichotomous variable. All patients in the cohort were designated either 'invited' or 'not invited'. Eligibility for CR was determined on the basis of hospital discharge cardiac diagnosis codes consistent with evidence-based guidelines for CR. The JHH CR program was sufficiently well resourced to accept all eligible patients and there were no other comparable CR programs available in the local area. Patient and clinician influences not captured in the study data were assumed to have had negligible impact on invitation. Ethics approval for the study was granted by the University of Newcastle and the Hunter New England Area Health Service.

The data set for the analysis comprised records linked between the John Hunter Hospital Cardiac Rehabilitation (JHH CR) cohort, which was established for research into the effectiveness of the JHH CR program, and the Admitted Patient Data Collection (APDC) which is the official database of inpatient statistics and includes information on all hospitalisations in the state of NSW. The JHH CR cohort combined information from a population-based regional cardiovascular disease (CVD) register and the JHH CR program database. Patients were eligible for the cohort in accordance with 'index hospitalisations' which were defined as 'first register recorded hospitalisations with specified coded cardiac inpatient diagnoses'. The intention was to crudely identify patients at a similar early stage of CHD.

The record linkage allowed consideration of social and demographic characteristics as well as factors related to index hospitalisations. There was no information on aspects of socioeconomic status (SES) such as occupation or education, and health insurance status was not a reliable indicator of SES. However in order to describe SES, postcode-based indices were appended to patient records in accordance with the Australian Bureau of Statistics (ABS) Census of Population and Housing 1996 Index of Relative Social Disadvantage (IRSD). This Index included area-based measures of social and economic characteristics, such as income, educational attainment and employment [11,12].

Patients were eligible for the study if separation dates for their index cardiac hospitalisations at JHH occurred between 1 July 1996 and 31 December 2000 inclusive and if they were aged between 20 and 84 years of age during their index hospitalisation. Travel distance was considered to have been a possible complicating factor. Therefore only patients who lived within the boundaries of the Lake Macquarie and Newcastle Local Government Areas, adjacent to the JHH, were included. Patients known to have not been invited for legitimate reasons, such as being too ill, awaiting further investigation or surgery, were excluded. Individuals who died in hospital during their index hospitalisation were included because some invited patients died in hospital.

Factors commonly reported in the literature as determinants of referral to hospital outpatient CR programs include: age, sex, marital status, measures of ethnicity and SES, cardiac events, hospital procedures and cardiac risk factors [13-29]. Many of these factors were included in the linked data set and were therefore considered to have been possible influences on invitation.

All clinical codes were based on the International Classification of Diseases and Related Health Problems Tenth Revision Australian Modification. Primary diagnosis field number 1 was used to assign patients into one of four possible cardiac groupings, which were AMI, UAP, HF or ischemic heart disease (IHD). This categorisation defined the index diagnosis variable. Codes in non primary, or 'secondary', diagnosis field numbers 2 to 20 were also reviewed. This was done so that variables denoting the presence or absence of UAP, IHD, HF or other cardiac conditions in any secondary diagnosis field could be added to patient records as a crude marker of disease severity.

Secondary diagnosis fields 2 to 20 were also used to identify the presence or absence of coded cardiac risk factors such as hypertension, smoking, hypercholesterolaemia, diabetes and depression. In addition these fields were used to identify the presence or absence of coded comorbidity, such as other vascular disease, malignant cancers, respiratory disease, arthropathies and end stage renal disease, that may have influenced invitation or attendance. Procedure codes were used to identify whether angiography, angioplasty/stenting or coronary artery bypass surgery had been undertaken during index hospitalisation.

Table 1 shows the factors upon which univariate tests were undertaken to ascertain statistical significance (P < 0.25) for inclusion in the multivariate model. Year of hospitalisation was included to allow for year to year variation in CR selection processes. Patients with index hospitalisations in 1996 or 1997 had similar odds of being invited to CR, while patients with index hospitalisations in 1998, 1999 or 2000, were twice as likely to have been invited, this being consistent with program changes in 1998. Hospitalisation year was re-classified as a dichotomy being 1996 or 1997, compared with 1998, 1999 or 2000. Hospital billing status classified patients in accordance with the way in which their stay was charged by the hospital i.e. public; private; Department of Veterans Affairs (DVA) or Workers Compensation (WC). While in some jurisdictions hospital billing status is associated with ability to pay, this did not apply here. High and low SES groups had roughly equivalent chances of being billed as 'public' patients.

**Table 1:** Possible factors associated with invitation to CR

Hospital related	Patient related	Indications in Secondary diagnoses fields
Hospitalisation year	Sex	UAP
Hospital billing status	Age	IHD
Index cardiac diagnosis	Marital status	HF
Length of hospital stay	Main language spoken	Other cardiac conditions
Hospital deaths	Region of Birth	Vascular disease
	Indigenous status	Malignant cancers
	IRSD	Respiratory disease
		Arthropathies
	Travel distance	End stage renal disease
	Admission type	Hypertension
		Smoker
		Hypercholesterolaemia
		Diabetes
		Depression
		Angiography
		Angioplasty/stenting
		Bypass surgery

**Note: **The APDC was the primary data source. The APDC is the official inclusive state-wide data collection of hospital inpatient statistics in NSW. The IRSD refers to Index of Relative Socioeconomic Disadvantage; UAP refers to unstable angina pectoris; IHD refers to ischaemic heart disease; HF refers to heart failure; main language spoken and region of birth were proxies for ethnicity.

The index cardiac diagnosis was included as a four group categorical variable, AMI, UAP, HF or IHD. The reference category for index diagnosis was AMI because all patients with a confirmed AMI diagnosis were routinely assessed for CR. Length of hospital stay was measured in days and was a significant predictor of invitation. There was no evidence of linearity between length of stay and invitation and length of stay was transformed and fitted as a polynomial. The hospital deaths variable was a significant predictor of invitation and was therefore retained.

Patients' age and sex were included as significant predictors of invitation. There was no evidence of linearity between age and invitation. Age was also transformed and fitted as a polynomial. Sex was a dichotomous variable. The APDC categorised patients who self-reported being either married/partnered never married/single; widowed; divorced or permanently separated. Due to small frequencies in the cells, marital status was re-grouped as a dichotomy, denoting patients known to be married or partnered, and patients known to not be married or partnered. Patients whose marital status was not known were excluded (n = 35). All patients admitted to NSW hospitals were asked to nominate their main spoken language as well as their country of birth. Region of birth was correlated with main language spoken. However region of birth was not a statistically significant predictor of invitation and was not included. Main language spoken was expressed as an English non-English dichotomy, this being the most suitable available proxy for ethnicity in this data set. Very few patients (n = 13) nominated Indigenous status, and due to unreliability in the data, Indigenous status was not included. The IRSD was re-grouped as a dichotomous variable, higher scores being associated with relative SES advantage. Travel distance, which measured road distance from patients' homes to the JHH, and type of admission (emergency or planned) were not statistically significant factors and were therefore not included in the multivariate model.

Binary variables derived from secondary diagnosis codes to denote the presence or absence of cardiac risk factors, additional cardiac diagnoses and co-morbidities, were included and further tested. Variables indicating that angiography, angioplasty/stenting or coronary artery bypass surgery had been conducted were also included. Multivariate logistic regression, at 5% level of significance and 80% power, tested the extent to which individual patient characteristics were statistically significantly associated with the binary outcome, invitation.

## Results

The patient cohort (n = 2,375) comprised 63% males (n = 1,503) and 37% females (n = 872). Just under half (49% or 1,170) of the patients were invited to the CR program. Of the invited patients, 69% were males, 31% were females and 70% were married or partnered. The median age was 69 years; 67 years for males compared with 72 years for females. Approximately 30% of all patients (and also men and women separately) had a length of hospital stay under five days; 35% had a length of stay between five and seven days (inclusive) and 35% stayed in hospital eight days or more.

Table 2 shows the fully adjusted model that best specified the influence of all statistically significant variables together on the linearity of the log odds of invitation to CR. Variables excluded from the adjusted model because they were not statistically significant in the multivariate analysis (P > 0.05) were: hypercholesterolaemia; vascular disease; respiratory disease; arthropathies; renal disease; hypertension; smoking; diabetes and depression. Secondary diagnoses for UAP, IHD and other cardiac conditions were not statistically significant predictors of invitation.

**Table 2:** Adjusted multivariate logistic regression of factors associated with invitation to CR (n = 2,375)

VARIABLES	N (%)	N INVITED to CR (%)	ODDS RATIO: 95% CONFIDENCE INTERVAL
**TOTAL**	2,375	1,170 (49.3%)	NA
**Sex**	2,375		
Males	1,503 (63%)	807 (69%)	1.00
Females	872 (37%)	363 (31%)	0.60 (0.39 to 0.92)
**Age**			
Age	2,375	NA	0.997 (0.996 0.998)
**Marital status**	2,340		
Not married	820 (35%)	339 (30%)	1.00
Married	1,520 (65%)	808 (70%)	1.34 (1.05 to 1.71)
**Main language spoken**	2,357		
Non-English	76 (3%)	20 (2%)	1.00
English	2,281 (97%)	1,138 (98%)	3.20 (1.63 to 6.32)
**IRSD**	2,355		
Less advantaged	634 (27%)	326 (28%)	1.00
More advantaged	1,721 (73%)	835 (72%)	0.71 (0.52 to 0.97)
**Hospitalisation year**	2,375		
1996 & 1997	857 (36%)	315 (27%)	1.00
1998, 1999 & 2000	1,518 (64%)	855 (73%)	3.61 (2.83 to 4.59)
**Hospital billing status**	2,373		
Public	1,759 (74%)	859 (73%)	1.00
Private	443 (19%)	241 (21%)	1.44 (1.07 to 1.93)
Other (WC, DVA)	171 (7%)	70 (6%)	1.66 (1.06 to 2.60)
**Index cardiac diagnosis**	2,375		
AMI	894 (38%)	632 (54%)	1.00
UAP	975 (41%)	326 (28%)	0.07 (0.06 to 0.10)
IHD	324 (14%)	195 (17%)	0.09 (0.06 to 0.13)
HF	182 (8%)	17 (2%)	0.04 (0.02 to 0.06)
**Length of hospital stay**	2,375		
Part 1		NA	402.20 (63.44 to 2549.91)
Part 2		NA	0.07 (0.03 to 0.16)
**Hospital deaths**	2,375		
Alive	2,263 (95%)	1,162 (99%)	1.00
Dead	112 (5%)	8 (1%)	0.05 (0.02 to 0.11)
**Bypass surgery**	2,375		
No	1,985 (84%)	870 (74%)	1.00
Yes	390 (16%)	300 (26%)	9.23 (5.96 to 14.30)
**Angiography**	2,375		
No	1,889 (80%)	842 (72%)	1.00
Yes	486 (20%)	328 (28%)	1.57 (1.14 to 2.17)
**Angioplasty/stenting**	2,375		
No	2,051 (86%)	968 (83%)	1.00
Yes	324 (14%)	202 (17%)	4.54 (3.08 to 6.68)
**HF as a secondary diagnosis**	2,375		
No	2,095 (88%)	1,076 (92%)	1.00
Yes	280 (12%)	94 (8%)	0.55 (0.38 to 0.80)
**Malignant cancer present**	2,375		
No	2,315 (98%)	1,150 (98%)	1.00
*Yes*	60 (3%)	20 (2%)	0.41 (0.20 to 0.83)

**Note: **The APDC was the primary data source. The APDC is the official inclusive state-wide data collection of hospital inpatient statistics in NSW. The IRSD refers to Index of Relative Socioeconomic Disadvantage; UAP refers to unstable angina pectoris; IHD refers to ischaemic heart disease; HF refers to heart failure; main language spoken and region of birth were proxies for ethnicity. Age was transformed as a one part polynomial expression. Length of stay was transformed as a two part polynomial expression.

Missing values for marital status (35), main language spoken (18), socioeconomic status (20) and billing status (2). Married/not married refers to those known to be married or partnered, compared with those known to be not married or partnered.

NA not applicable.

Factors that were statistically significant predictors of invitation in the adjusted model (P < 0.05) were: sex; age; marital status; main language spoken; IRSD; hospitalisation year; hospital billing status; index cardiac diagnosis; length of hospital stay; hospital deaths; bypass surgery; angiography; angioplasty/stenting; heart failure as a secondary diagnosis, and malignant cancer. Being married, having English as the main spoken language, or undergoing bypass surgery, angiography, or angioplasty/stenting at index hospitalisation, were factors which were positively associated with invitation. Being female, older, relatively more socioeconomically advantaged, having a longer length of hospital stay, or a secondary diagnosis of heart failure or malignant cancer at index hospitalisation, were all negatively associated with invitation. Patients who died in hospital on their index stay were less likely to have been invited to CR. Compared with patients hospitalized in 1996 or 1997, patients with index hospitalisations in 1998, 1999, or 2000 were more likely to have been invited to CR. Compared with publicly billed patients, privately billed patients and patients billed as WC or DVA were more likely to have been invited to CR. Patients with an index cardiac diagnosis of AMI were more likely to have been invited to CR, compared with patients with an index cardiac diagnosis of UAP, IHD or HF.

Females had 40% lower odds of being invited, compared with males, and married patients had 30% higher odds of being invited, compared with not married patients. Patients whose preferred language was English had three times the odds of being invited compared with those for whom English was not the preferred language. Based on the IRSD, more advantaged patients had approximately 30% lower odds of being invited, compared with relatively disadvantaged patients. Older patients were less likely to have been invited compared with younger patients, and patients with longer lengths of hospital stay were less likely to have been invited, compared with those with shorter hospital stays. Plots of age and invitation and length of stay and invitation showed these patterns.

## Discussion

Australian and international studies have reported CR referral rates of less than 50% amongst eligible populations [21-24,26,28]. Consistent with the literature, this study showed that males had increased odds of being invited, as did younger patients, patients who were known to be married, and patients from English speaking backgrounds [15,17,23-26,28]. Possible explanations for the inequalities are the fact that females in the cohort may have been judged as being less suitable for CR because they were older than males, but there may have been other explanations that were not evident. A statistical decomposition method, used to explain the sex inequality in invitation in these data, is the subject of a separate publication [30].

Cardiac rehabilitation programs were originally established to facilitate early return to work and this may have been a reason why younger males (with higher expected remaining working years) had higher odds of being invited, although this could not be tested here because data on patients' employment or occupational status were not available [21,31]. Social support is seen as an important factor in the rehabilitation of patients with cardiac disease and there is evidence that patients with relatively higher levels of social support are more likely to benefit from CR [19,20,32,33]. The finding that married or partnered patients were preferentially invited may have been related to assumed associations between marriage, social support and CR participation.

The IRSD was the main SES measure included here. The finding that relatively disadvantaged groups were more likely to have been invited to CR suggested that SES inequalities were not an equity issue. However the underlying SES distribution within this particular population of hospital inpatients may not have been representative of the broader population. Of primary importance from an equity perspective is the finding that, after allowing for other known 'reasonable' factors, individuals were being invited to CR on the basis of individual social and demographic characteristics such as sex, age, marital status and main language spoken.

The result that, compared with patients hospitalized in 1996 or 1997, patients with index hospitalisations in 1998, 1999, or 2000 were more likely to have been invited to CR, may have been related to an expansion of the JHH CR program which occurred in 1998. Compared with publicly billed patients, patients who were billed as private, WC or DVA were more likely to have been invited to CR, although the reasons for this were not clear from the results.

## Limitations

It is regrettable that data on ethnicity were not available for this study. Main language spoken was a proxy for ethnicity although statistical power was limited by the small percentage of patients reporting a main language other than English (3%). It is also unfortunate that Indigenous status is poorly reported in Australian hospitals and was not sufficiently reliable for testing. However the finding that relatively disadvantaged groups were more likely to have been invited to CR was not generally consistent with the literature which shows that disadvantaged groups are less likely to be referred to CR [13,25,34]. In spite of possible limitations, the area-based IRSD was a suitable indicator of SES for Australian hospital data.

All patients in the study were eligible for CR assessment on the basis of primary hospital discharge codes. Although we were able to use secondary diagnosis codes to ascertain the extent of illness related factors that may have influenced invitation, we were unable to accurately determine the level of disease severity, cardiac risk and comorbidity in the cohort. It is therefore possible that invitation may have been influenced by factors not tested here, although the degree to which this may have occurred is not known from these data.

There is evidence to show that clinicians can influence referral to CR [27,35,36] and the fact that clinician data were not available for this study was a limitation. One study which used a hierarchical design to investigate clinician and patient factors that influenced CR referral, found that both clinicians and patients were relevant contributors [29]. It is possible that patient and clinician related factors not included here, may have been proxies for other relevant factors or may have confounded the results in ways that were not obvious from this analysis [21]. There may have also been patient and provider needs and preferences that were not taken into account in this study. Clearly more work is needed to better understand issues related to needs, preferences and communication in healthcare [4,37-41].

Although the intention of the study was to demonstrate an approach, the results need to be cautiously interpreted, given the time that the data were collected and the subsequent increased funding for the management of chronic disease in Australia. Change in access to cardiac rehabilitation over time is the subject of ongoing research.

## Conclusions

The purpose of the paper was to demonstrate a way of assessing equity of access opportunities through an analysis of linked records from a population source, an inpatient statistics collection and a local service database. Achieving fairness and justice in the distribution of health opportunities is necessary for equity in health. Better ways of informing health services policy and decision makers about inequalities and inequities in patient selection processes are clearly needed. The approach demonstrated here has practical implications for health service clinicians, managers and other providers. System factors can and do influence equity of access to healthcare.

## Competing interests

The authors declare that they have no competing interests.

## Authors' contributions

JSW undertook this work as part of doctoral degree at the University of Newcastle, Australia under the academic supervision of JB and KI. JSW conducted the statistical analyses and drafted the manuscript. The final version was approved by all authors.
